# Annealing temperature and environment effects on ZnO nanocrystals embedded in SiO_2_: a photoluminescence and TEM study

**DOI:** 10.1186/1556-276X-8-517

**Published:** 2013-12-06

**Authors:** Kantisara Pita, Pierre Baudin, Quang Vinh Vu, Roy Aad, Christophe Couteau, Gilles Lérondel

**Affiliations:** 1 School of Electrical and Electronic Engineering, Nanyang Technological University (NTU), Block S2, Nanyang Avenue, Singapore 639798, Singapore; 2 CINTRA, CNRS-NTU-Thales UMI 3288, Research Techno Plaza, 50 Nanyang Drive, Border X Block, Level 6, Singapore 637553, Singapore; 3 Laboratory for Nanotechnology, Instrumentation and Optics (LNIO), University of Technology of Troyes (UTT), 12 rue Marie Curie, Troyes 10000, France

**Keywords:** ZnO nanocrystals, Photoluminescence, UV emission

## Abstract

We report on efficient ZnO nanocrystal (ZnO-NC) emission in the near-UV region. We show that luminescence from ZnO nanocrystals embedded in a SiO_2_ matrix can vary significantly as a function of the annealing temperature from 450°C to 700°C. We manage to correlate the emission of the ZnO nanocrystals embedded in SiO_2_ thin films with transmission electron microscopy images in order to optimize the fabrication process. Emission can be explained using two main contributions, near-band-edge emission (UV range) and defect-related emissions (visible). Both contributions over 500°C are found to be size dependent in intensity due to a decrease of the absorption cross section. For the smallest-size nanocrystals, UV emission can only be accounted for using a blueshifted UV contribution as compared to the ZnO band gap. In order to further optimize the emission properties, we have studied different annealing atmospheres under oxygen and under argon gas. We conclude that a softer annealing temperature at 450°C but with longer annealing time under oxygen is the most preferable scenario in order to improve near-UV emission of the ZnO nanocrystals embedded in an SiO_2_ matrix.

## Background

Recently, ZnO nanocrystals (ZnO-NCs) have attracted a lot of interests because of their promising applications in optoelectronic devices, such as light-emitting devices or UV photodetectors [1,2]. The near-UV emission of ZnO-NC can also be utilized for efficient energy transfer to rare earth ions (e.g., Eu^3+^ and Er^3+^ ions) to obtain emission in the visible (for lighting) or in the near-infrared (for telecommunications) regions [3,4]. In order to facilitate the energy transfer, the emission band from the excited ZnO must overlap with the absorption band of the rare earth ions. In our earlier work [3], for example, the ZnO films were doped with Cd ions to maximize the overlap between the emission of Cd-doped ZnO and the absorption of Eu^3+^ ions. We propose here the development and study of ZnO-NC embedded in a SiO_2_ matrix to have a broadband near-UV emission from ZnO to facilitate and optimize the energy transfer to rare earth ions without introducing doping ions such as Cd ions [3]. It is desirable to embed ZnO-NCs in a dielectric matrix, such as SiO_2_, to provide both chemical and physical protection for the ZnO-NCs [5] and also to incorporate rare earth ions.

Many existing studies have already intensively reported on the various fabrication techniques and optical properties of ZnO-NCs embedded in SiO_2_[5-15]. Nonetheless, a complete investigation on the growth of ZnO-NCs as a function of annealing temperature under different annealing environments is essential to understand the influence of various annealing conditions on the optical properties of ZnO-NC:SiO_2_ systems. Through this understanding, the emission of ZnO-NCs can be engineered to provide optimum energy transfer to rare earth ions as mentioned above. We report in this article the study on optical and structural properties of ZnO nanocrystals embedded in SiO_2_ matrix using the low-cost sol–gel technique. We show that annealing temperature and annealing atmosphere are crucial parameters that can be optimized in order to maximize the near-UV emission from the ZnO-NCs. Transmission electron microscopy (TEM) images as well as photoluminescence (PL) spectra are studied in order to find the right conditions for obtaining a maximized emission. A blueshifted emission at 360 nm was necessary to account for the emission of the smallest-size NCs. Such a result is in agreement with earlier-reported blueshifted transmission spectra observed for ZnO-NCs but diluted in solution, not in thin films [16].

## Methods

**Figure 1:**
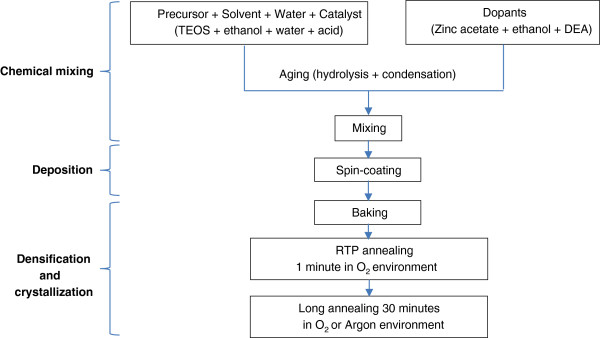
**The fabrication of ZnO nanocrystals embedded in SiO**_**2**_**matrix by the low-cost sol–gel technique.**

We have developed a low-cost fabrication process to prepare our composite thin film samples using the sol–gel technique. The process consists of three steps, as shown schematically in Figure 1. The first step is mixing the precursors, solvent, and catalysts. Tetraethyl orthosilicate (TEOS) and zinc acetate were used for SiO_2_ and ZnO precursors, respectively. TEOS was mixed with ethanol, and then a controlled amount of deionized (DI) water and acid was added. Zinc acetate was mixed in ethanol and diethanolamine (DEA). The ratio of ZnO to SiO_2_ (ZnO/SiO_2_ = 1:2 in this article) is determined by controlling the amount of the precursors in the sols. The sols are aged at an appropriate time, typically 24 h, to form Si-O-Si and Zn-O networks. The two sols are mixed together before the second step. The second step is to spin-coat the sol on (100) Si wafer substrates. This step is followed by soft baking for 5 min at 100°C and then rapid thermal processing (RTP) annealing for 1 min in an O_2_ environment at various annealing temperatures ranging from 450°C to 700°C. To investigate the emission from ZnO nanocrystals, the samples were post-annealed for 30 min in O_2_ and Ar environments at various temperatures.

## Results and discussion

### TEM of ZnO nanocrystals embedded in SiO_2_ matrix

**Figure 2:**
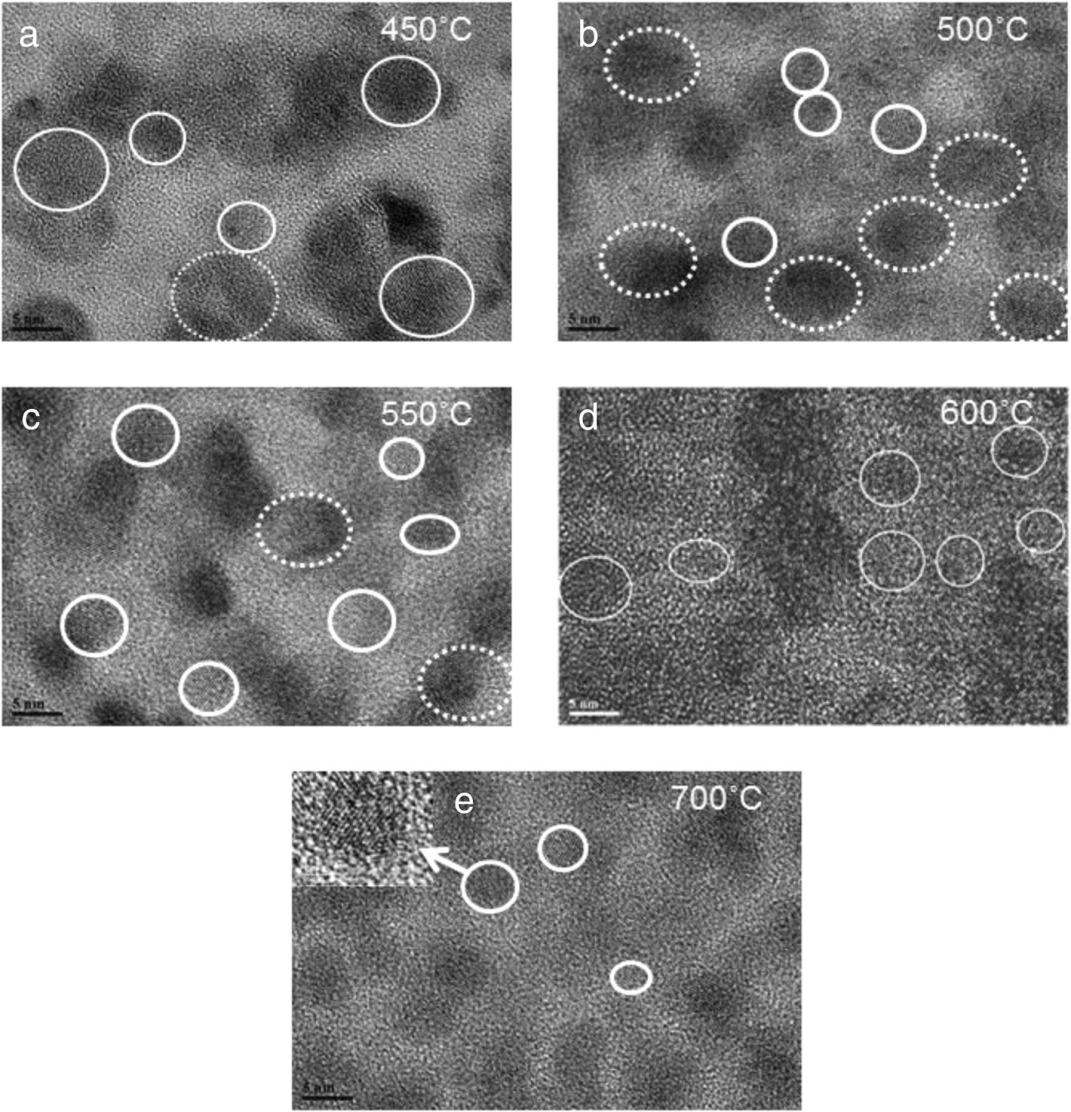
**TEM pictures of samples annealed in RTP for 1 min in O**_**2 **_**atmosphere. (a)** 450°C, **(b)** 500°C, **(c)** 550°C, **(d)** 600°C, and **(e)** 700°C.

As mentioned in the 'Introduction,’ in order to study the formation and evolution of ZnO-NCs in a SiO_2_ matrix at various annealing temperatures and environments, we have employed the TEM technique and analysis. Figure 2a,b,c,d,e shows the TEM pictures of the samples annealed in RTP for 1 min in O_2_ atmosphere at 450°C to 700°C. The ZnO nanocrystals in the SiO_2_ matrix can be identified by the presence of crystal planes which are indicated by white circles. The dark contrast indicates the presence of ZnO clusters. From the TEM pictures in Figure 2a,b,c,d,e, we obtained the average sizes of the ZnO-NCs and their standard deviations for various RTP annealing temperatures, presented in Table 1. We can verify that the atomic spacing found by the TEM images is indeed that of the ZnO crystals. We see that the average sizes and the standard deviations decrease with increasing temperature. The decrease of the average sizes of ZnO-NCs with increasing annealing temperature is presumably because of the formation of Zn_2_SiO_4_ at the ZnO and SiO_2_ interfaces [6]. The reduction of the corresponding standard deviation indicates that the average sizes become more uniform with increasing temperature.

**Table 1:** Average sizes and corresponding standard deviations of the ZnO-NCs for various annealing temperatures

**Temperature (°C)**	**Average size (nm)**	**Standard deviation (nm)**
450	4.83	1.51
500	4.22	1.60
550	4.14	1.12
600	3.91	0.85
700	3.13	0.48

### Photoluminescence of ZnO-NCs in SiO_2_ at various annealing temperatures

**Figure 3:**
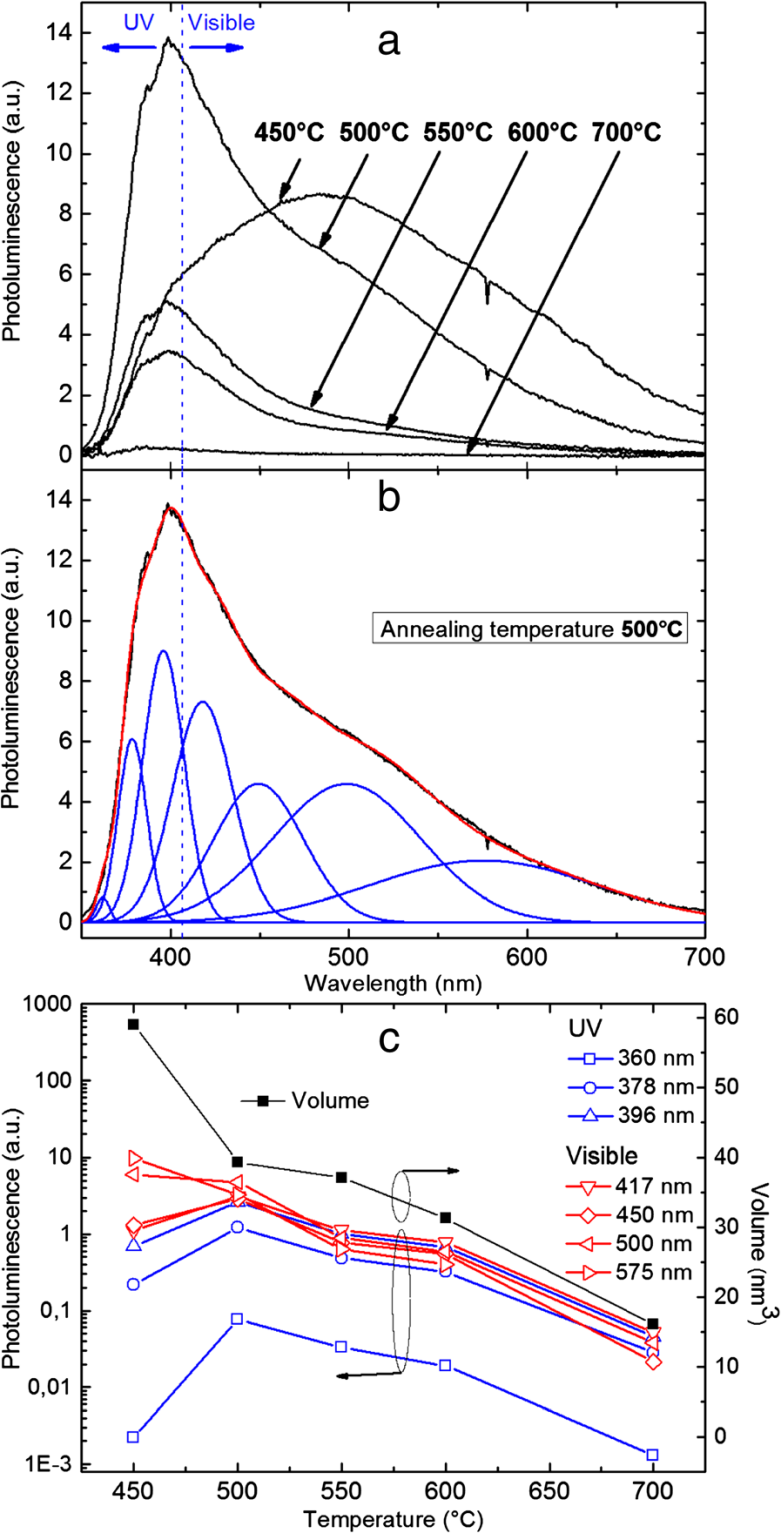
**The PL spectra of the samples at various temperatures. (a)** Photoluminescence spectra of the ZnO**-**NCs in the SiO_2_matrix at various RTP annealing temperatures. **(b)** The spectrum can be accounted for by two main contributions in the UV-blue and visible regions, respectively. **(c)** The evolution of various peaks as a function of annealing temperature is shown. For comparison, the volume evolution calculated from the NC size obtained from the TEM analysis is also shown. The decrease of the signal at high annealing temperature can be roughly accounted for by the decrease of the NC absorption cross section.

The emission from the ZnO-NCs in the SiO_2_ matrix at various RTP annealing temperatures was investigated using PL with a 325-nm He-Cd continuous excitation laser. Emission was sent to a 50-cm focal length spectrometer coupled to a Peltier-cooled CCD camera at -85°C. The PL spectra are shown in Figure 3a for various RTP annealing temperatures. As shown in Figure 3b for the most representative spectrum, the measured PL can be perfectly accounted for using two main contributions, one in the UV-blue range and the other one in the visible range. The UV-blue emission is composed of three Gaussian peaks centered at 360, 378, and 396 nm. The visible emission is composed of four Gaussian peaks centered at 417, 450, 500, and 575 nm. The photoluminescence from our SiO_2_ matrix alone was measured beforehand and was found to be negligible as no emission could be detected under our experimental conditions. To further confirm the consistency of the emissions, the same analysis has been performed for all spectra, keeping the fitting parameters the same except for the peak amplitude, i.e., fixed center wavelengths and full width at half maxima were used for all spectra. Figure 3c shows the evolution of the area of each Gaussian peak as a function of the RTP temperature, along with the evolution of the ZnO-NC average volume. The average ZnO-NC volume is determined using the average size of the ZnO-NC given in Table 1 and by assuming that the ZnO-NCs have a spherical shape. At 450°C annealing temperature, the PL spectrum (Figure 3a) is very broad and is centered at about 500 nm. As seen in Figure 3c, the PL spectrum is mainly constituted by the Gaussian peaks around 500 and 575 nm. The visible ZnO emission is due to defects in the sample which can be attributed to the great number of ZnO clusters and the relatively poor ZnO-NC crystallinity, especially at the ZnO-NC/SiO_2_ interface, as seen in the TEM image (Figure 2a). The ZnO defects are mainly oxygen-related defects. The emission at 417 nm can be assigned to oxygen interstitials [17], while the other visible emissions at 450, 500, and 575 nm can be related to oxygen vacancies [5,13,18]. These defects are consistent with our long annealing data, which will be discussed in the next section.

On the other hand, the few ZnO-NCs that exist in the sample give rise to some UV emission, which results in the broad PL spectrum. At 500°C annealing temperature, the PL spectrum exhibits an overall blueshift which is due to the increase of the UV-blue emission in the sample. As shown in Figure 3c, the RTP annealing at 500°C is accompanied by an increase of the blue and UV emission between 360 and 450 nm and a decrease of defect emissions at higher wavelengths. The drastic change in the emission spectrum of the sample can be attributed to an increase in the ZnO-NCs and the decrease of ZnO clusters in the sample (Figure 2b), which should in turn increase the ZnO near-band-edge emission in the UV region. The emission peak at 378 nm can be related to ZnO near-band-edge (excitonic) emission [19,20]. The emission peak at 396 nm could possibly be related to the electron transition from Zn interstitial to Zn vacancy as reported by Panigrahi et al*.*[5]. While being relatively weak, it is worth noting the appearance of a peak at 360 nm for the smallest NCs for which quantum confinement is expected to occur as already reported in a transmission experiment in solution [16]. Further analysis and especially low-temperature PL measurement are needed to confirm the peak origin. For annealing temperatures higher than 550°C, no drastic change is observed in the shape of the emission spectra, as seen in Figure 3a. Instead, the PL spectra mainly exhibit a decrease in the emission intensity. Indeed the Gaussian fitting analysis shows that the peak amplitudes decreased by the same proportion compared to its value at 500°C. However, the analysis shows that the decrease of the defect emission is slightly stronger than that of the UV emission contribution. The overall decrease of the emission intensity is consistent with the reduction of the ZnO-NC average volume (i.e., size) with increasing annealing temperature, as shown in Figure 3c. The decrease of the ZnO-NC average volume normally results in a decrease of the ZnO-NC absorption cross section, leading to a weaker ZnO-NC luminescence.

### Photoluminescence of ZnO-NCs in SiO_2_ after the second annealing step in O_2_ or Ar atmosphere

**Figure 4:**
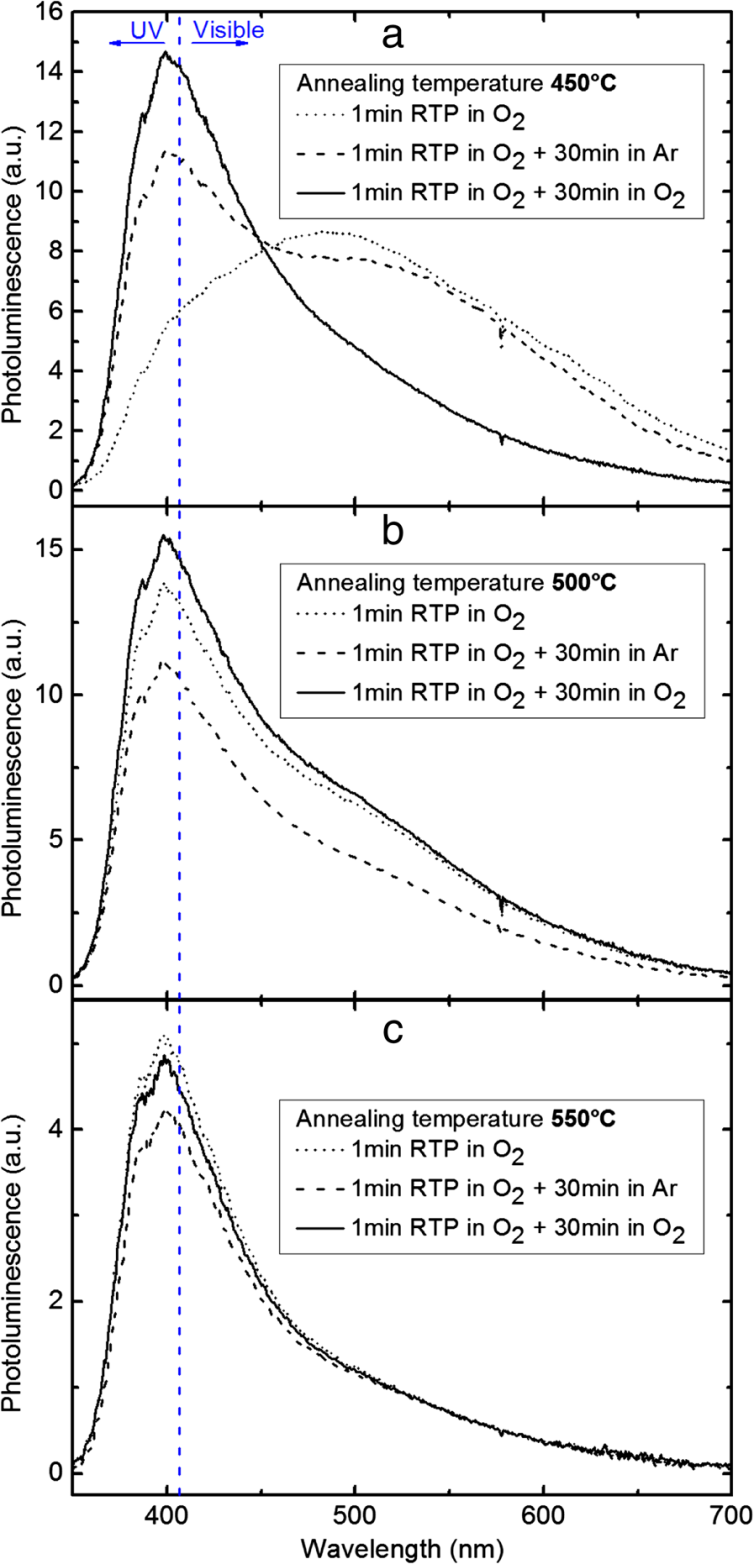
**PL of samples going through the second annealing step in O**_**2 **_**and Ar atmospheres.** At **(a)** 450°C, **(b)** 500°C, and **(c)** at 550°C.

The RTP-annealed samples at 450°C, 500°C, and 550°C were post-annealed for 30 min in both O_2_ and Ar atmospheres. The PL spectra are shown in Figure 4a,b,c. The post-annealing process was not realized for the samples annealed in RTP beyond 550°C as they presented a very weak emission.

For the sample annealed in RTP at 450°C, the PL spectra (see Figure 4a) show a remarkable change in the emission characterized by a decrease of the defect (i.e., visible) emission and the appearance of the UV emission around 378 and 396 nm. Compared to the post-annealing in Ar, the post-annealing in O_2_ results in a stronger decrease of the defect emission around 500 and 575 nm. This behavior strongly indicates that oxygen vacancies are at the origin of the defect emissions in the visible region, which supports our analysis above that the defects are due to the oxygen vacancies. For the samples annealed in RTP at 500°C, the PL spectra present a slight change in the shape of the emission. Nonetheless, the post-annealing in Ar results in an overall decrease of the emission intensity, while the post-annealing in O_2_ leads to an increase in the UV emission and a comparatively slight decrease in the defect emissions. The slight decrease in the defect emissions indicated that the RTP annealing at 500°C for 1 min is sufficient to form the ZnO-NC and significantly reduces the oxygen deficiency. For the sample annealed in RTP at 550°C, the post-annealing in Ar and O_2_ hardly presents any change in the emission spectra, except for a slight change in the intensity of the UV emission. The post-annealing in Ar and O_2_ has no effect on the sample after the RTP annealing at 550°C.

## Conclusions

To conclude, we studied ZnO nanocrystals embedded in SiO_2_ matrix fabricated by the sol–gel method. We have analyzed the effects of temperature and atmosphere on the annealing of such thin films. We post-annealed the samples from 450°C to 700°C under O_2_ or under Ar atmosphere. By looking at the effect of such annealing conditions using TEM images and PL spectra, we identify the best annealing temperature for maximizing the near-UV emission of the ZnO nanocrystals. We show that an annealing temperature of 450°C under longer annealing time and under oxygen is preferable to higher annealing temperatures and shorter times. By maximizing the near-UV emission of the ZnO nanocrystals, which produce a relatively wide emission band centered at ~398 nm, the spectral overlapping with rare earth ions like Eu^3+^ (which has an absorption band at 395 nm) can be greatly enhanced. These results are important in the process of making efficient luminescent thin films (including energy transfer to other species such as rare earth ions) for future applications in lighting and telecommunication based on ZnO-NCs.

## Competing interests

The authors declare that they have no competing interests.

## Authors’ contributions

KP initiated and supervised the research work as well as started the write-up. PB carried out the experimental work and analyzed the data. QVV participated in the studies and prepared and improved the manuscript. RA worked on the simulation of PL data. CC participated in the studies and improved and prepared the manuscript for submission and publication. GL participated in the studies, initiated the simulation of PL data, and improved the manuscript. All authors read and approved the final manuscript.
